# The Polymorphisms with Cataract Susceptibility Impair the EPHA2 Receptor Stability and Its Cytoprotective Function

**DOI:** 10.1155/2015/401894

**Published:** 2015-11-19

**Authors:** Jin Yang, Dan Li, Qi Fan, Lei Cai, Xiaodi Qiu, Peng Zhou, Yi Lu

**Affiliations:** ^1^Department of Ophthalmology, Eye and ENT Hospital, Fudan University, 83 Fenyang Road, Shanghai 200031, China; ^2^Myopia Key Laboratory of Health PR China, Shanghai 200031, China; ^3^Research Center, Eye and ENT Hospital, Fudan University, 83 Fenyang Road, Shanghai 200031, China; ^4^Department of Ophthalmology, Parkway Health Hong Qiao Medical Center, Shanghai 200336, China

## Abstract

Despite accumulating evidence revealing susceptibility genes for age-related cataract, its pathophysiology leading to visual impairment at the cellular and molecular level remains poorly understood. Recent bioinformatic studies uncovered the association of two single nucleotide polymorphisms in human EPHA2, rs2291806 and rs1058371, with age-related cataract. Here we investigated the role of EPHA2 in counteracting oxidative stress-induced apoptosis of lens epithelial cells. The cataract-associated missense mutations resulted in the destabilization of EPHA2 receptor without altering the mRNA transcription. The cytoprotective and antiapoptotic function of EPHA2 in lens epithelial cells was abolished by the functional polymorphisms. Furthermore, our results suggest that the downstream signaling of activated EPHA2 promotes the antioxidative capacity of lens epithelial cells to eradicate the overproduction of reactive oxygen species. In contrast, the overexpression of EPHA2 with nonsynonymous mutations in the lens epithelial cells offered limited antioxidative protection against oxidative stress. Thus, our study not only sheds the light on the potential cytoprotective function of EPHA2 signaling in lens but also provides the cellular mechanisms underlying the pathogenesis of age-related cataract.

## 1. Introduction

Cataract, the opacity of crystalline lens, is the leading cause of blindness and visual impairment worldwide. While congenital cataract is largely inherited in a Mendelian manner with high penetrance, both genetic risk and environmental factors contribute to age-related cataract [1, 2]. Cumulative damage of environmental insults exerts oxidative stress on lens epithelial cells with genetic susceptibility and induces cellular apoptosis, a common cellular mechanism underpinning noncongenital cataract [3–5]. Recent genetic and epidemiological studies suggest the association of Eph-receptor tyrosine kinase-type A2 (EPHA2) with human age-related cataract in distinct populations [6–9]. Despite the bioinformatic screening of nonsynonymous single nucleotide polymorphism (SNP) in* EPHA2* gene as potential risk variants for cataract [10], the cellular and molecular mechanisms underlying its pathogenesis remain elusive.

As a member of the Eph superfamily of receptor tyrosine kinases, the forward signaling cascade of EPHA2 is primarily mediated by its corresponding ephrin-A ligands [11, 12]. Genetic and pharmacological inhibition of EPHA2 induces apoptosis and abrogates tumorigenic growth of tumor cells [13–16]. EPHA2 protein is expressed in human and mouse lens [6], implying its potential role in maintaining lens clarity during aging by promoting cell viability. The combined application of bioinformatic tools including Soft Intolerant from Tolerant (SIFT), Polymorphism Phenotype (PolyPhen), and I-Mutant identified nonsynonymous rs2291806 and rs1058371 as potential functional polymorphisms [10].

The accrual of oxidative damage to lens epithelial cells at least partially causes age-related cataract [17–19]. Under physiological conditions, reactive oxygen species (ROS) are scavenged and eliminated by superoxide dismutase (SOD) in the mitochondria [20]. Either the overproduction of ROS or the dysfunction of endogenous antioxidants disrupts the redox homeostasis and thus triggers the apoptotic process and pathogenesis of the disease [21, 22]. It was shown that the induction of ROS activated EPHA2 receptor to promote virus entry during Kaposi's sarcoma-associated herpesvirus (KSHV) infection [23], which raises questions about the antioxidant role of EHPA2 signaling. Here we show the cytoprotective function by EPHA2 against oxidative stress-mediated damages as well as the antioxidative capacity of EPHA2 polymorphisms rs2291806 and rs1058371 which predispose the individuals to age-related cataract.

## 2. Materials and Methods

### 2.1. Cell Culture

The human lens epithelial cell (HLEC) line SRA 01/04 (transformed by Simian virus 40 large T antigen) was purchased from the American Type Culture Collection [24]. For maintenance, HLECs were cultured in Dulbecco's modified eagle's medium (DMEM; Invitrogen, Carlsbad, CA, USA) with 10% of heat inactivated fetal bovine serum (Invitrogen), 100 U/mL of penicillin (Sigma, St. Louis, MO, USA), and 100 *μ*g/mL of streptomycin (Sigma) in humidified 5% CO_2_ at 37°C.

### 2.2. Plasmid Construction and Cell Transfection

The wild-type human* EPHA2* gene (NM_004431.3) was generated by PCR using the following primers:

 forward primer: 5′-CTAGCTAGCATGGAGCTCCAGGCAGCCCGC-3′,
 reverse primer: 5′-ACGCGTCGACTCAGATGGGGATCCCCACAGT-3′.

The PCR product was then subcloned into Ubi-MCS-3FLAG-SV40-EGFP-IRES-puromycin lentiviral vector. The plasmids encoding EPHA2 polymorphism: rs1058371 (286A>T; forward primer: 5′-GAGGCTGAGCGTATCTTCTTTGAGCTCAAGTTTACTG-3′, and reversed primer: 5′-CAGTAAACTTGAGCTCAAAGAAGATACGCTCAGCCTC-3′), rs2291806 (2473G>A; forward primer: 5′-TGGGAGTTGTCCAACCACAAGGTGATGAAAGCCATCA-3′, and reversed primer: 5′-TGATGGCTTTCATCACCTTGTGGTTGGACAACTCCCA-3′), and rs3754334 (2874C>T; forward primer: 5′-CGGCCACCAGAAGCGCATTGCCTACAGCCTGCTGGGA-3′, and reversed primer: 5′-TCCCAGCAGGCTGTAGGCAATGCGCTTCTGGTGGCCG-3′), were constructed using Multipoints Mutagenesis Kit (Takara, Dalian, Liaoning, China). The lentiviral plasmid encoding WT or mutated EPHA2 was cotransfected with pMDL, pRev, and pVSVG into 293gp cells to generate high-titers of lentivirus, followed by ultracentrifugation of viral supernatants [25]. HLECs were infected with diluted lentivirus and the green fluorescence signal was examined under a fluorescence light microscope (Olympus Inc., Tokyo, Japan) with digital images captured.

### 2.3. Real-Time PCR

Total RNA was extracted with Trizol reagent (Invitrogen) from the HLECs 72 h after infection. First strand cDNAs were synthesized from 1.0 *μ*g total RNA by reverse transcription using the RevertAid H Minus First Strand cDNA Synthesis Kit (Hanover, MD, USA). Real-time PCR was performed using the following primers: forward primer 5′-TGGCTCACACACCCGTATG-3′ and reversed primer 5′-GTCGCCAGACATCACGTTG-3′. As an internal control, *β*-*actin* was amplified using 5′-CATTAAGGAGAAGCTGTGCT-3′ and 5′-GTTGAAGGTAGTTTCGTGGA-3′ as forward and reverse primers, respectively.

### 2.4. Western Blotting Analysis

Cell lysates were collected from the HLECs 72 h after infection with lentivirus expressing wild-type EPHA2 or mutants. The protein concentration was determined by BCA assay. The proteins were separated by electrophoresis and transferred to a nitrocellulose membrane. The blocked membrane was incubated overnight with primary antibodies against EPHA2, GFP, or GAPDH (Santa Cruz, CA, USA) at 4°C. Following washing three times in TBST, the membrane was incubated with goat anti-mouse HRP-conjugated secondary antibodies for 30 min at room temperature. After washes with TBST, immunoreactive signals were detected using enhanced chemiluminescence reagent (Pierce). Images were captured with the ChemiDocTMMP imaging system (Bio-Rad, Hercules, CA, USA). The densitometric intensity of the imaged bands was analyzed by Image-Pro Plus 5.0 (Media Cybernetics, Silver Spring, EUA). Triplicate experiments were performed.

### 2.5. Cell Viability Assay

Cell viability and proliferation were determined using a Cell Counting Kit-8 (CCK-8) assay (Dojindo Laboratories, Kumamoto, Japan), which is a sensitive measurement of the survival status of cells. HLECs in the logarithmic growth phase were collected and seeded in 96-well plates (1 × 10^4^ cells/well). After culture in the absence of antibiotics for 24 h, cells were infected with lentivirus encoding wild-type EPHA2 or SNP mutants for 72 h. To each well, 100 *μ*L CCK8 solution dissolved in DMEM was added. After incubation for 3 h, the optical density of formazan crystals was measured in an X Mark microplate spectrophotometer (Bio-Rad) at 450 nm. Eight duplicate wells were used for measurement. Triplicate experiments were performed.

### 2.6. Measurement of Lipid Peroxidation Products

Lipid peroxidation was assessed with malondialdehyde (MDA) assay using Lipid Peroxidation MDA Assay Kit from Beyotime. Briefly, HLECs with lentiviral infection were lysed in 0.1 M Tris/HCl buffer (pH 7.4 containing 0.5% Triton X-100, 5 mM *β*-mercaptoethanol, and 0.1 mg/mL PMSF) 72 h after transfection. The lysate supernatant (0.1 mL) was mixed with trichloroacetic acid (15%, w/vol), thiobarbituric acid (0.375%, w/vol), and hydrochloric acid (0.25 M) at a 1 : 1 : 1 : 1 ratio. The mixture was heated at 100°C for 30 min, immediately cooled, and then centrifuged (3,500 ×g for 5 min). The absorbance of the supernatant was measured at 532 nm. The amount of thiobarbituric-acid-reacting substance (TBARS) was calculated MDA equivalents as previously described [26]. Triplicate experiments were performed.

### 2.7. Superoxide Dismutase (SOD) Activity Assay

In this assay, a water-soluble formazan dye is produced from WST-1 upon its reduction by superoxide anion. The rate of the superoxide anion-mediated reduction is linearly related to the xanthine oxidase activity and is inhibited by SOD, and the inhibitory activity of SOD can be determined by a colorimetric method. To perform this assay, HLECs were seeded on 6-well plates, infected with lentivirus, and lysed in ice-cold 0.1 M Tris/HCl buffer 72 h later. The lysates were centrifuged at 14000 ×g at 4°C for 5 min and the supernatant was collected. The SOD activity in the supernatants was determined by measuring the absorbance at 450 nm in a spectrophotometer. Triplicate experiments were performed.

### 2.8. Total Antioxidant Capacity (TAC) Assay

Total Antioxidant Capacity (TAC) Colorimetric Assay Kit from BioVision was used to measure the endogenous antioxidants. Briefly, HLECs cells were infected with lentivirus in 6-well plates and collected with ice-cold 0.1 M PBS. Cell lysates were centrifuged at 14000 ×g at 4°C for 4 min and the supernatant was harvested. The absorbance of the supernatant was measured at 570 nm in an X Mark microplate spectrophotometer (Bio-Rad). Triplicate experiments were performed.

### 2.9. Flow Cytometric Detection of Apoptosis Assay

Apoptosis was evaluated by APC-annexin V/7-AAD (BD Pharmingen, California, USA) staining followed by flow cytometric analysis. Cells were plated in 6-well plates at a density of 1 × 10^5^/well and cultured for 48 h with reagents. Then, the cells were gently trypsinized and washed twice with ice-cold PBS. At least 10,000 cells were resuspended in 100 *μ*L 1x binding buffer, stained with 5 *μ*L 7-AAD (25 *μ*g/mL) and 5 *μ*L APC-annexin V at 4°C for 30 min, and immediately analyzed with a FACScanto flow cytometer (BD Bioscience, USA). Each measurement was carried out in triplicate.

### 2.10. Statistical Analysis

Data were expressed as mean ± standard error and analyzed by one-way ANOVA and* post hoc* Bonferroni's test. The statistical software, Prism 5 (GraphPad software Inc., San Diego, CA, USA), was used. The criterion for statistical significance was *p* < 0.05.

## 3. Results

### 3.1. Impaired Protein Stability Caused by Missense Mutation in EPHA2

**Figure 1:**
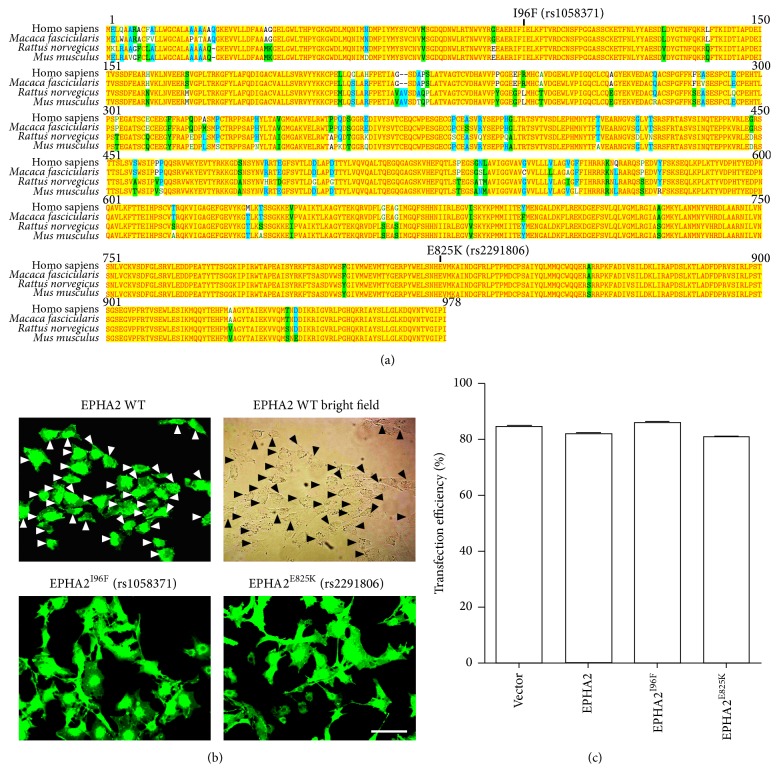
The evolutional conservation of cataract-associated SNP and the overexpression of EPHA2 in lens epithelial cells. (a) The protein sequences of human, macaque, rat, and mouse EPHA2 were aligned for multiple comparison. The functional polymorphisms rs1085371 and rs2291806 are highlighted here to show the missense mutation in EPHA2. The sequence alignment shows the evolutional conservation of both amino acids with substitution. (b) The human lens epithelial cells (HLECs) were infected with lentivirus encoding wild-type EPHA2, EPHA2^I96F^, or EPHA2^E825K^. The images captured with fluorescent microscopy 72 h after infection are shown here. Scale bar: 50 *μ*m. (c) The infection efficiency was quantified by counting GFP-positive cells and total number of cells. Data are shown as the mean ± SEM.

There are a total of 134 nonsynonymous SNPs identified within the coding region of* EPHA2* gene. A previous bioinformatic analysis suggests that rs2291806 (E825K) and rs1058371 (I96F) are potential functional polymorphisms involved in susceptibility to cataract formation [10]. Multiple sequence alignment of human, macaque, rat, and mouse EPHA2 showed that both amino acids with missense mutations are evolutionarily conserved (Figure 1(a)). To investigate the cytoprotective role of EPHA2 in lens epithelial cells and the molecular mechanism underlying the association of EPHA2 mutation with age-related cataract, we generated the lentiviral plasmids encoding wild-type EPHA2, EPHA2^E825K^, and EPHA2^I96F^ and prepared the high-titers of corresponding lentivirus for infection. The human lens epithelial cells (HLECs) were subsequently infected with lentivirus expressing either wild-type or mutant EPHA2. The quantification by counting the green fluorescent protein- (GFP-) positive cells showed that ~85% of cells on average were overexpressed by EPHA2 and its missense mutants 72 h after infection (Figures 1(b) and 1(c)).

**Figure 2:**
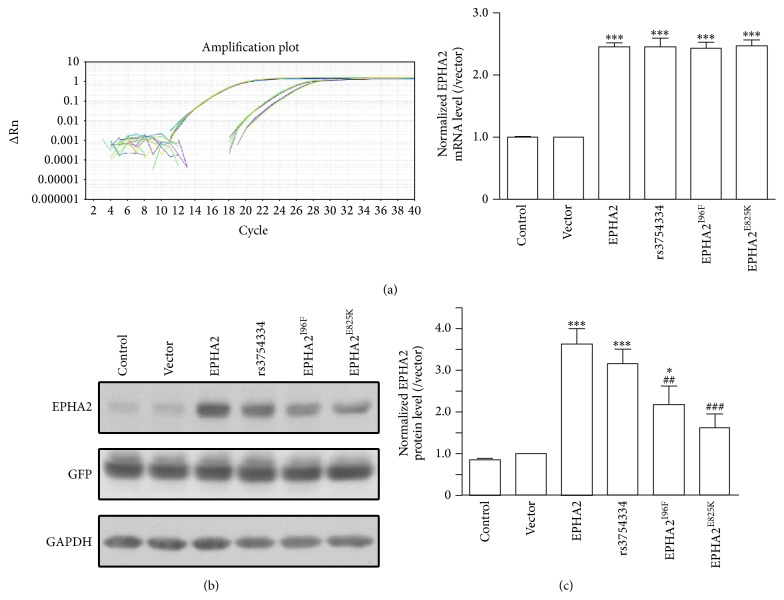
The protein destabilization caused by cataract-associated missense mutation. (a) The* EPHA2* mRNA transcriptional level was measured with real-time PCR after HLECs were overexpressed with wild-type or mutant EPHA2 for 48 h. The* EPHA2* mRNA level was normalized with the internal control *β*-actin and the data are presented as ratio of vector control. (b) The EPHA2 protein expression was tested with western blotting after the lentivirus-mediated overexpression in HLECs. (c) Quantitative analysis shows the impaired protein stability of EPHA2 with missense mutation. The EPHA2 protein level was normalized with the internal control* GAPDH*. Data are shown as the mean ± SEM. ^*∗*^ *p* < 0.05, ^*∗∗∗*^ *p* < 0.001 versus vector control; ^##^ *p* < 0.01, ^###^ *p* < 0.001 versus wild-type EPHA2.

The abundance of* EPHA2* mRNA in each group after lentiviral infection was evaluated with real-time PCR. There was a 2.5-fold increase in the level of* EPHA2* mRNA following lentivirus-mediated overexpression, as compared with noninfected cells and vector control (Figures 2(a) and 2(b)). Our previous study showed that a synonymous polymorphism within* EPHA2* gene, named rs3754334, is associated with the risk of age-related cataract [27]. Using the synonymous substitution as negative control, we compared the level of mRNA transcripts encoding the wild-type and mutant EPHA2 and found no difference in the transcription (Figure 2(b)).

The missense mutations with apparent effects on function are dominated by compromised protein stability [28]. Thus, we further examined the protein level of EPHA2 without or with amino acid substitution in HLECs by western blot. The results showed that EPHA2^I96F^ and EPHA2^E825K^ mutation significantly impaired the receptor stability while synonymous SNP rs3754334 did not affect the protein expression of EPHA2 (Figure 2(c)). Taken together, these results suggest that the missense mutations in EPHA2 are potentially associated with age-related cataract via compromising the macromolecular stability and its functional network.

### 3.2. Loss of Cytoprotective Function by Cataract-Associated Mutation in EPHA2

**Figure 3:**
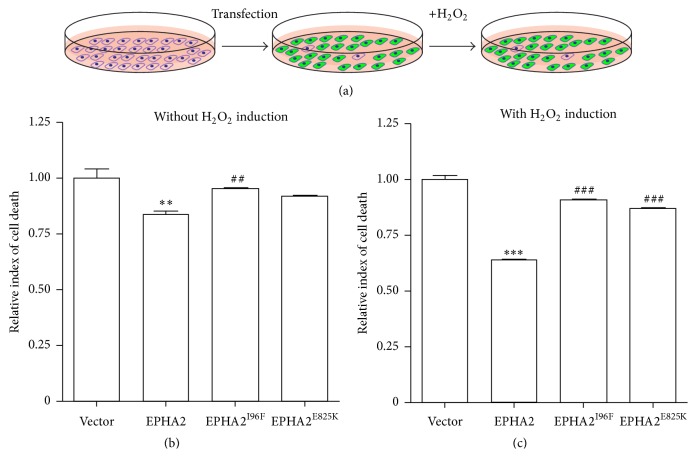
The functional polymorphisms abolish EPHA2-mediated cytoprotective effect. (a) The experimental paradigm. The HLECs were seeded and infected with lentivirus expressing wild-type EPHA2, EPHA2^I96F^, or EPHA2^E825K^. The infected cells were treated with 200 *μ*M H_2_O_2_ to mimic the light-induced oxidative stress in lens. (b-c) The cell viability was assayed before and after H_2_O_2_ treatment following lentivirus-based gene overexpression. The quantification reveals the cytoprotective effect of wild-type EPHA2 and the loss of function in cataract-associated mutants. Data are shown as the mean ± SEM. ^*∗∗*^ *p* < 0.01, ^*∗∗∗*^ *p* < 0.001 versus vector control; ^##^ *p* < 0.01, ^###^ *p* < 0.001 versus wild-type EPHA2.

Eph receptors have been shown to play critical roles in tissue boundary formation, neural crest cell migration, axon guidance, bone remodeling, and vascular organization [12]. However, whether the EPHA2 signaling pathway provides cytoprotective effects against oxidative challenge remains largely unclear. To decipher the cellular functions of EPHA2, we overexpressed wild-type EPHA2 in HLECs and examined the cell viability before and after the treatment with 200 *μ*M H_2_O_2_ to induce oxidative stress (Figure 3(a)). The relative index of cell death was evaluated by

(1) Absorbance  index=The  number  of  starter  cellsAbsorbance  value×10000,Relative  index=Absorbance  index  of  experimental  groupAvergae  absorbance  index  of  vector  group.

The absorbance value at 450 nm was determined using CCK8 assay. The relative results of cell viability assay without H_2_O_2_ stimulation imply that EPHA2 overexpression may enhance the proliferation of lens epithelial cells (Figure 3(b)). The quantitative analysis also showed that overexpression of EPHA2 in HLECs reduced the relative index of cell death and especially ameliorated H_2_O_2_-induced apoptosis (Figures 3(b) and 3(c)), suggesting the cytoprotective function of EPHA2 in the cultured lens epithelial cells. In addition, we found that the cytoprotective effect on oxidative stress was abolished by either EPHA2^I96F^ or EPHA2^E825K^ mutation (Figure 3(c)).

**Figure 4:**
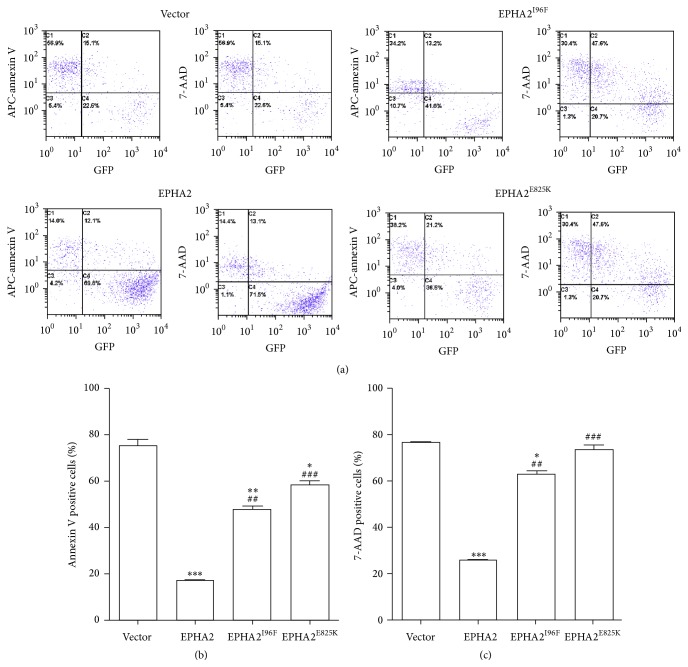
Compromised antiapoptotic function in cataract-associated genetic mutants. (a) After lentiviral infection to introduce control vector, EPHA2, EPHA2^I96F^, or EPHA2^E825K^ into the cells and the administration with 200 *μ*M H_2_O_2_, the HLECs were digested, dissociated, and stained with either APC-annexin V or 7-AAD, followed by the fluorescence activated cell sorting to analyze the early and late apoptosis. The GFP signal intensity was compromised if the HLECs undergo oxidative stress-induced apoptosis. (b-c) The statistical analysis shows that overexpression of wild-type EPHA2 reduces the proportion of both early and late apoptotic cells. The missense mutations abolish the antiapoptotic effect against oxidative damage. ^*∗*^ *p* < 0.05, ^*∗∗*^ *p* < 0.01, and ^*∗∗∗*^ *p* < 0.001 versus vector control; ^##^ *p* < 0.01, ^###^ *p* < 0.001 versus wild-type EPHA2.

To specifically determine the antiapoptotic effect of EPHA2 in HLECs in vitro, we further performed the staining with APC-annexin V or vital dye 7-AAD on the dissociated cells, followed by fluorescent flow cytometry to analyze the proportion of H_2_O_2_-induced early and apoptotic cells. The data indicated that HLECs with wild-type EPHA2 overexpression displayed a striking reduction in both early and late apoptosis, as compared with control cells undergoing cell death together with the diminishing GFP signal (Figure 4). As expected, ectopic expression of either EPHA2^I96F^ or EPHA2^E825K^ in HLECs showed reduced cytoprotective effects against H_2_O_2_-induced cell death (Figure 4). These data combined demonstrated the protective function of EPHA2 in lens epithelial cells through preventing apoptosis and the neutralization of antiapoptotic effect by two functional polymorphisms.

### 3.3. Abrogated Antioxidative Effect by Nonsynonymous Polymorphisms with Risk of Cataract

**Figure 5:**
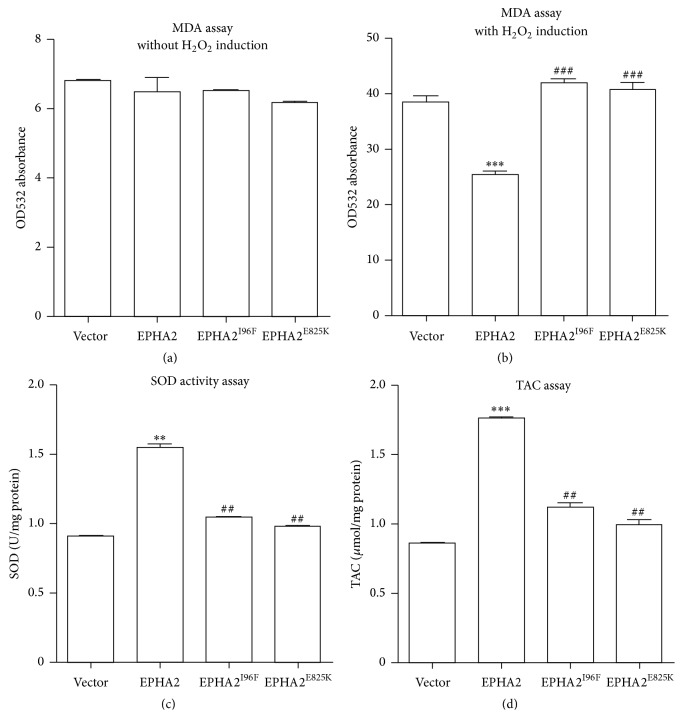
The nonsynonymous polymorphisms nullify the antioxidative capacity of EPHA2. (a-b) The lipid oxidation was evaluated with MDA assays in the HLECs before and after H_2_O_2_ treatment. The overexpression of EPHA2 reduces the absorbance value of cell lysate with H_2_O_2_ treatment, while the introduction of EPHA2^I96F^ or EPHA2^E825K^ mutant does not decline the lipid oxidation. (c-d) The SOD activity and the total antioxidative capacity were tested with SOD and TAC assay kit, respectively. The data are presented as U/mg or *μ*mol/mg proteins. The upregulation of SOD level and total antioxidant content by EPHA2 is abrogated by the cataract-associated SNPs. ^*∗*^ *p* < 0.05, ^*∗∗*^ *p* < 0.01, and ^*∗∗∗*^ *p* < 0.001 versus vector control; ^##^ *p* < 0.01, ^###^ *p* < 0.001 compared with wild-type EPHA2.

The ROS generated endogenously or induced by environmental stress have long been implicated in cell death and tissue injury in the context of age-related cataract [3–5, 17, 18, 22]. The most efficient enzymatic antioxidants in the lens include SOD, catalase, glutathione peroxidase, and cytosolic glutathion-S-transferase [22, 29]. To investigate the mechanisms underlying the antiapoptotic effect of EPHA2 in the lens, we assessed the levels of lipid peroxidation in the HLECs by MDA assay. Despite the undetectable effect by EPHA2 overexpression under basal conditions, we found that the activation of EPHA2 signaling significantly declined the H_2_O_2_-induced oxidation of lipids in the lens epithelial cells (Figures 5(a) and 5(b)). Moreover, the SOD activity and the total antioxidative potency were upregulated by overexpression of EPHA2 to counterbalance the production of ROS in HLECs (Figures 5(c) and 5(d)). Interestingly, the antioxidative effect of EPHA2 was consistently abrogated by the identified functional polymorphisms rs2291806 and rs1058371 (Figure 5). Our results showed the antioxidative role of EPHA2 in the lens epithelial cells under the exposure of extrinsic oxidative stress.

## 4. Discussion

Although genetic studies have hitherto provided a deep insight into the understanding of genetic framework involved in the age-related cataract, its pathophysiology remains to be elucidated. The putative role of EPHA2 in the etiopathogenesis of age-related cataract has attracted much attention regarding the molecular mechanisms involved in maintaining the clarity of lens by EPHA2. Here we report that EPHA2 signaling protects the lens epithelial cells from oxidative stress-induced cell death. Furthermore, the loss of protein stability in two of the nonsynonymous polymorphisms compromises the antioxidative and antiapoptotic effect of EPHA2.

The previously identified cataract-associated mutations in EPHA2 basically reside in kinase and sterile alpha motif (SAM) domains [2, 12]. It is hypothesized that the loss of EPHA2 function may directly or indirectly impair cellular structural stability, cell-to-cell crosstalk, protein folding, and transcriptional activation, which cause congenital or age-related cataract [6, 12]. Impaired development of lens fiber cells or equatorial cells caused by loss of function in EPHA2 may lead to hereditary cataract, whereas accumulating oxidative stress resulting from both environmental insults and age-dependent reduction of EPHA2 expression in lens could contribute to age-related cataract [6, 12, 17, 30, 31]. Our data suggests that EPHA2 plays a cytoprotective role in lens epithelial cells by promoting cell viability under oxidative stress. In spite of the established regulatory role of Eph/ephrin role in the epithelial morphogenesis and homeostasis [32], we did not find conspicuous differences in the morphology of lens epithelial cells between control and overexpressing cells. Consistent with bioinformatic analysis showing that both rs2291806 and rs1058371 are the least stable among varieties of SNPs [10], our biochemical data confirms that both functional polymorphisms in EPHA2 lead to the destabilization of the receptor and thus neutralize its antiapoptotic role. The decay of mutant EPHA2 is possibly caused by reduced protein solubility and ubiquitin-mediated proteasomal degradation [33]. These results further support that the genetic mutation-mediated protein degradation contributes to apoptosis in age-related degenerative diseases [34, 35].

We further identified the antioxidative effect of EPHA2 by gain-of-function analysis, which underlies its cytoprotective function against environmental insult. Activation of Nrf2 increases the elimination of both exogenous and endogenous toxic chemicals including ROS and Nrf2-dependant signaling regulates the gene expression of EPHA2 [36, 37]. Indeed, our data revealed the upregulation of antioxidative SOD activity and the neutralization of lipid oxidation by EPHA2 activation in the lens epithelial cells. However, the complex transcriptional activation mediated by overexpression of EPHA2 to increase antioxidative capacity still remains to be determined. Importantly, the missense mutations in EPHA2 associated with age-related cataract abolished the EPHA2-mediated enhancement of antioxidative capacity, suggesting the loss of function in both polymorphisms.

## 5. Conclusion

In summary, our study revealed the cytoprotective and antioxidative function of EPHA2 in lens epithelial cells, which coordinate and maintain the lens epithelial structural integrity. The nonsynonymous polymorphisms rs2291806 and rs1058371 disrupt the protein stability and diminish the antiapoptotic effect of EPHA2 in human lens during aging, contributing to age-related cataract. The bioinformatic prediction helps us to identify the functional SNP in disease. The future gain- and loss-of-function studies in the animal model will further elucidate the cytoprotective and antioxidative role of EPHA2 in vivo.
